# C-Reactive Protein and Coronary Heart Disease: All Said—Is Not It?

**DOI:** 10.1155/2014/757123

**Published:** 2014-04-07

**Authors:** Frederik Strang, Heribert Schunkert

**Affiliations:** ^1^Medizinische Klinik II, Universitätsklinikum Schleswig-Holstein, UKSH Campus Lübeck, Ratzeburger Allee 160, 23562 Lübeck, Germany; ^2^Klinik für Herz- und Kreislauferkrankungen, Deutsches Herzzentrum München, Technische Universität München, Lazarettstraße 36, 80636 München, Germany

## Abstract

C-reactive protein (CRP) and coronary heart disease (CHD) have been the subject of intensive investigations over the last decades. Epidemiological studies have shown an association between moderately elevated CRP levels and incident CHD whereas genetic studies have shown that polymorphisms associated with elevated CRP levels do not increase the risk of ischemic vascular disease, suggesting that CRP might be a bystander rather than a causal factor in the progress of atherosclerosis. Beside all those epidemiological and genetic studies, the experimental investigations also try to reveal the role of CRP in the progress of atherosclerosis. This review will highlight the complex results of genomic, epidemiological, and experimental studies on CRP and will show why further studies investigating the relationship between CRP and atherosclerosis might be needed.

## 1. Introduction

**Figure 1:**
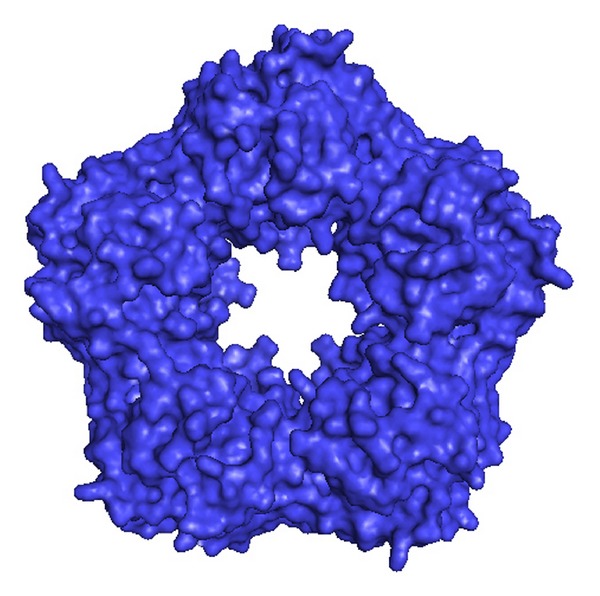
The picture shows the C-reactive protein as it circulates in the human blood stream after response to inflammatory stimuli. The five subunits are forming a disc-shaped pentamer around a central pore. (Picture was generated with information from the RCSB Protein Data Bank by using PyMOL software.)

C-reactive protein (CRP) circulates as a disc-shaped pentamer consisting of five identical subunits arranged around a pore in the middle (Figure 1). Measurements of its serum concentrations are used clinically as unspecific marker for inflammation. As the exact function of CRP is not fully understood yet, it is believed that it functions as part of the innate immune system [1]. It is also known that CRP rises in severe unstable angina and has a prognostic value [2]. Specifically high CRP levels following myocardial infarction are associated with adverse outcomes, including left ventricular failure [3], and increased rates in cardiac death and ventricle rupture [4, 5]. Massive data collected over the past decades showed an association between moderately elevated CRP levels and incident coronary heart disease (CHD) [6–9]. In 2008 a genetics study investigated the question of whether polymorphisms in the CRP gene are associated with increased levels of CRP, thereby offering an instrument for studying the causality of CRP in the risk of coronary heart disease [10] trying to answer the chicken or egg question [11]. This study came to the conclusion that genetically elevated CRP levels do not increase the risk of ischemic vascular disease, suggesting that CRP might be a bystander rather than a causal factor in the progress of atherosclerosis. In parallel to this disappointing data biochemical studies opened the opportunity that monomeric CRP—rather than the pentamer—may play a functional role in CHD. This review will highlight the complex results of genomic, epidemiological, and experimental studies on CRP and will show why further studies investigating the relationship between CRP and atherosclerosis might be needed.

## 2. C-Reactive Protein

### 2.1. The Structure of Human CRP

CRP circulates in the human serum as a noncovalently bound disc-shaped pentamer consisting of five identical subunits [12]. It presents two faces: a binding side where it binds calcium-dependent to its widely recognized specific ligands and an effector side. Each subunit consists of 206 amino acids with a molecular weight of 23 kDa and carries 2 calcium ions essential for the pentameric isoform [13]. Under physiological circumstances, that is, calcium present in the extracellular environment and a physiological pH, it remains quite stable unless it binds to one of its specific ligands [14]. It has a particularly high affinity for phospholipids, especially lysophospholipids [15] found on the surface of damaged or apoptotic cells [16]. Upon binding to one of its ligands it dissociates into monomers [17, 18].

### 2.2. The Function of Human CRP

Since neither a deficiency of CRP is known nor a therapeutical inhibitor has yet been tested* in vivo,* the role of CRP in physiological or disease settings remains elusive. As it binds to phospholipids, especially lysophospholipids, and recognizes bacterial lipids, it has been suggested that it functions as part of the innate immune system. Once CRP has bound to one of its ligands and dissociated into its monomers, it presents properties not shared with the circulating pentameric CRP. The pentameric CRP appears to have no interaction with complement or the regulatory complement factor H [19] whereas the monomeric CRP can directly activate the complement cascade through C1q fixation [20] and induce platelet [21] and monocyte activation [22].

### 2.3. The Synthesis of Human CRP

CRP is mostly synthesised in the liver—although extrahepatic transcription of CRP has been described [23–25]—upon inflammatory stimuli as interleukin-1, interleukin-6, and tumor necrosis factor *α* [12]. It can rise from baseline to its 10,000-fold upon bacterial inflammation. Following myocardial infarction, an increase in CRP levels is also observed [26]. This response is very quick and happens within 12 hours reaching its peak at about 50 hours after stimuli [27]. Actually, it seems that most forms of adverse stress are associated with an increase in CRP levels [28] (see also review [12]).

## 3. C-Reactive Protein and Coronary Heart Disease

### 3.1. Epidemiological Studies

Since the first epidemiological study describing an association between elevated CRP levels and an increased risk for CHD events was published, more than 50 studies have followed [29]. In a meta-analysis published in the year 2004 by Danesh et al. 22 of those studies were included [30]. Those 22 studies involved 7068 cases of coronary heart disease with a mean followup of 12 years and presented an overall odds ratio of 1.58 (95 percent confidence interval, 1.48 to 1.68) among patients with values from the top third compared with the bottom third of baseline C-reactive protein concentrations. These results were quite similar to a subanalysis of the four biggest studies on 4107 cases of coronary heart disease, which provided an odds ratio of 1.49 (95 percent confidence interval, 1.37 to 1.62). Five years later another meta-analysis was published involving 23 studies with 8 more recent articles presenting an overall odds ratio of 1.60 (95 percent confidence interval, 1.43 to 1.78) by comparing CRP levels of <1.0 mL/dL versus >3.0 mL/dL showing no major difference to the studies published before [31].

Studies like those mentioned before implied that CRP levels can be used to reclassify subjects who fall into the intermediate-risk category of CHD in 2008, introducing the Reynolds Risk Score for man and woman [32, 33]. The scores improved accuracy of clinical algorithms for global cardiovascular risk prediction that reclassified subjects at intermediate-risk into higher- or lower-risk categories. Another score based on serum levels of CRP, fibrin degradation products, and heat shock protein 70 as predictors of future risk of death and myocardial infarction in patients with suspected or known CHD followed in 2013 [34].

In 2005 a statin therapy trial comparing moderate statin therapy (40 mg pravastatin daily) and intensive statin therapy (80 mg atorvastatin daily) for patients with CHD showed that a decrease in CRP levels during statin treatment independently and significantly correlates with progression of atherosclerosis [35]. Another study followed in 2008: the JUPITER trial [36]. JUPITER enrolled 17,802 individuals without manifest cardiovascular disease. All participants had low-density lipoprotein cholesterol (LDLC) levels below 130 mg/dL, but CRP levels greater than 2 mg/dL. The subjects were randomly designated to rosuvastatin 20 mg daily or placebo. The trial was stopped early because the interim results met the study's predefined stopping criteria by showing a 44 percent reduction in the trial primary endpoint of all vascular events. The placebo event rate in this study indicates that elevated CRP levels have high vascular risk even when LDLC levels lie within the range of current guidelines, being consistent with meta-analyses of CRP and CHD mentioned before.

Neither the meta-analyses nor the JUPITER trial were able to answer the question if CRP is a causal factor in coronary heart disease or just an innocent bystander in inflammation, a well- accepted pathomechanism in atherosclerosis [8, 37]. But several recent studies showed a significant contribution of CRP to coronary risk prediction independent of the traditional Framingham Risk Score [38] and therefore it found its way into clinical guidelines for guidance of measures in primary prevention [39].

### 3.2. Experimental Studies

#### 3.2.1. Pentameric CRP

CRP has been an object of experimental studies over the last three decades. There have been reports suggesting prothrombotic, proinflammatory, and proatherogenic properties* in vivo *for native CRP. Particularly since it was shown that CRP can activate the classical complement cascade, it was most likely that it would have proinflammatory properties in general [40, 41]. As CRP has been found in atherosclerotic plaques [42], it would have been a perfect explanation for the results from the epidemiological studies. But further studies were not always able to reproduce the above-mentioned properties, so that the initial results were most likely due to the commercial preparation of CRP with remaining toxic sodium acid or presence of bacterial endotoxin (lipopolysaccharide) in CRP produced by recombinant* Escherichia coli* [12, 43–45].

Not much less data exists for animal models trying to answer the question of a prothrombotic property of CRP [46–49]. With a single exception, these studies found no association between human CRP and a progression of atherosclerosis and it seemed more likely that CRP might act just as a bystander rather than a causal factor. But almost all of those studies used mice as an animal model for atherosclerosis which might have been the pitfall. The differences of CRP between species are enormous regarding ligand binding, secondary binding effects, and complement activation and behaviour as an acute phase reactant [29]. This is why Pepys et al. used a rat reperfusion model in 2006 to study tissue damage induced by human CRP following myocardial infarction and the ability to block the damage by a CRP inhibitor [50].

Even though it was shown that human CRP activates the classical complement cascade in humans and rats, it is not possible to explore those results of injecting human CRP into another species to the pathomechanism of the common clinical observation of increased CRP levels following myocardial infarction and the adverse outcomes.

#### 3.2.2. Monomeric CRP

With these controversial results an elegant solution to the conflicting CRP data was the introduction of the concept that monomeric forms of CRP might occur following the binding of circulating pentameric CRP to one of its ligands [14, 51], resulting in subsequent dissociation and functional activation. The existence of monomeric CRP is known over decades [52] and seemed to be a nonsoluble tissue-based protein rather than a soluble plasma-based protein, with antigenicity-expressing neoepitopes differing from native CRP epitopes [53]. This monomeric CRP has proinflammatory properties not shared with pentameric CRP like C1q fixation [14], to promote neutrophil-endothelial cell adhesion [54], platelet activation [55], thrombus formation [55, 56], and monocyte chemotaxis [17]—to name just a few. Thus the controversial results can be either the product of contaminated CRP (with toxic sodium acid or bacterial endotoxin) or the product of dissociated CRP.

Particularly, studies showed that the monomeric form of CRP—and not native CRP—colocalizes with complement in infarcted regions [57–60]. Since Molins et al. showed that monomeric but not pentameric CRP displays a prothrombotic phenotype enhancing not only platelet deposition, but also thrombus growth under arterial flow conditions, a possible role for monomeric CRP in the pathogenesis of “active” CHD needs to be considered [55]. Interestingly, about 1 year later Eisenhardt et al. showed the deposition of monomeric CRP in human aortic and carotid atherosclerotic plaques but not in healthy vessels [17]. The pentameric isoform was found neither in healthy nor in diseased vessels. In a study from 2012 Habersberger et al. showed that the nonsoluble monomeric CRP can be detected on microparticles from patients with acute myocardial infarction, whereas significantly less monomeric CRP was detectable on microparticles from healthy controls and stable CHD patients [51].

But unless clinical studies with a direct inhibitor of (monomeric) CRP can be conducted in human beings, the complex function of CRP can only be speculated.

### 3.3. Genetic Studies

Epidemiological studies showing an association between an exposure (in this case CRP) and a disease (here CHD) are sometimes confounded even with the most carefully study design. Genetic studies using a Mendelian randomization design utilize a method to estimate the causal nature of exposures and to avoid reverse association bias [61]. As quite a few studies have shown that multiple single nucleotide polymorphisms in the CRP gene (or the promoter region of the CRP gene [62, 63]) are associated with an increase in CRP baseline levels [64–66], it was just a matter of time for large genetic studies to follow estimating the association of elevated CRP levels and an increased risk for CHD [10, 67–69].

A first hint gave the population-based Rotterdam Study published in 2006. Kardys et al. analysed 5231 men and women for the association between CRP-related haplotypes and CHD. In contrast to epidemiological studies, such genetically elevated CRP levels were not found to be an independent marker of increased risk for CHD in patients without a history of CHD. Three haplotypes were identified as being associated with CRP levels, but the CRP haplotypes themselves were not associated with CHD.

Then a few more epidemiological studies followed involving over 28,000 CHD cases and 100,000 controls with none of them showing an association between elevated CRP levels and CHD [10, 68].

As a designated number of patients and controls are needed for Mendelian randomization studies the CRP, CHD Genetics Collaboration was founded in 2008. About three years later in 2011 the results were published including over 46,000 patients with prevalent or incident CHD and almost 150,000 controls. CRP variants were associated with up to 30% difference in CRP concentration per allele. Like all the genetic studies before, there was no association between single nucleotide polymorphisms associated with raised CRP levels and CHD so that most likely CRP is not even a modest causal factor in CHD.

The strong data from the genetic studies analysing an association between elevated CRP concentrations and the risk of CHD make it most doubtful that circulating pentameric CRP has a direct pathological role in the progression of CHD even though Mendelian randomizations also have limitations [70].

## 4. Conclusion

The data on CRP is massive and seems most controversial by trying to harmonize the results from genetic studies, epidemiological studies, and experimental investigations. The strong data from the Mendelian randomizations made it most unlikely that elevated CRP play a direct causal role in CHD. But that is consistent with experimental data as no prothrombotic or proinflammatory properties have been established for circulating CRP. The results showing proinflammatory and prothrombotic effects were most likely due to contamination of commercial CRP with either bacterial endotoxin, toxic sodium acid, or monomeric CRP. The probable tissue damage by CRP following myocardial infarction mediated by complement is most likely due to binding of CRP to apoptotic cells and subsequent dissociation to monomeric CRP which may have proinflammatory qualities like classical complement activation by complement fixation.

The epidemiological studies demonstrated an association between elevated CRP concentrations and an increased risk for CHD events in the first place. Meanwhile, with the results from the Mendelian randomization studies and the experimental data, elevated CRP in the epidemiological setting must be seen as a bystander rather than a causal factor of CHD. Nevertheless, it is undoubted that a prediction model that incorporates high-sensitivity CRP improves global cardiovascular risk prediction. The reduction of CHD events in patients with moderately elevated CRP levels and an intensive statin therapy can be the result of unknown anti-inflammatory property of statins and the subsequent decrease of CRP levels.

Thus, we should not stop our investigations at the “marker versus maker” debate on CRP but try to understand the inflammatory process associated with atherosclerosis. As pointed out before CRP is a downstream biomarker of elevated interleukin-1, interleukin-6, and tumor necrosis factor *α*. Interestingly, two Mendelian randomization studies showed that a genetic polymorphism in the interleukin-6 receptor signalling pathway associates with lower levels of CRP and a reduction of cardiovascular events [71, 72]. This data supports a causal association between inflammatory activation and atherosclerosis. With these data in mind we are looking forward to the results from the CANTOS and CRIT trials. In both studies the investigators are targeting inflammatory upstream pathways. In the Canakinumab Anti-inflammatory Thrombosis Outcomes Trial (CANTOS), a human monoclonal antibody (Canakinumab) that specifically inhibits IL-1*β* is tested to reduce recurrent vascular events whereas in the Cardiovascular Inflammation Reduction Trial (CIRT) methotrexate as a tumor necrosis factor *α* and interleukin-6 inhibitor is applied to postmyocardial infarction patients to examine the promising animal data showing a slowdown in atherosclerotic lesion progression in cholesterol-fed rabbits [73].

## 5. Perspective

As monomeric CRP has an effect on thrombus formation, the question would be if moderately elevated CRP levels are associated with an increased risk for CHD events in “active” CHD. Do instable plaques expose binding ligands to circulating pentameric CRP which can lead to CRP dissociation and induction of local inflammation? This question is answered neither by current data of genetic analyzes nor by current data of experimental approaches nor by current data of epidemiological studies.

An elegant way to evaluate a functional role of CRP in CHD would be randomized trial with a direct CRP inhibitor. With 1,6-bis-phosphocholine such compound was first tested in animal models. Another interesting approach is the antisense oligonucleotide ISIS-CRP_Rx_, which reduces the CRP production in the liver and is currently tested in a phase 2 study on patients with rheumatoid arthritis, according to the manufacturer's website with promising success. Noteworthy, in 2011 Wang et al. showed an aptamer binding specific to monomeric but not to pentameric CRP [74]. With an additional blocking quality we would have a new approach to directly distinguish between monomeric and pentameric effects [74]. We are most excited about the first results from* in vivo* applications from all of those approaches.
